# Unique properties of TCR-activated p38 are necessary for NFAT-dependent T-cell activation

**DOI:** 10.1371/journal.pbio.2004111

**Published:** 2018-01-22

**Authors:** Muhammad S. Alam, Matthias M. Gaida, Subrata Debnath, Harichandra D. Tagad, Lisa M. Miller Jenkins, Ettore Appella, M. Jubayer Rahman, Jonathan D. Ashwell

**Affiliations:** 1 Laboratory of Immune Cell Biology, National Cancer Institute, National Institutes of Health, Bethesda, Maryland, United States of America; 2 Institute of Pathology, University Hospital Heidelberg, Heidelberg, Germany; 3 Laboratory of Cell Biology, National Cancer Institute, National Institutes of Health, Bethesda, Maryland, United States of America; 4 Laboratory of Molecular Immunology at the Immunology Center, National Heart, Lung, and Blood Institute (NHLBI), National Institutes of Health (NIH), Bethesda, Maryland, United States of America; St. Jude Children’s Research Hospital, United States of America

## Abstract

Nuclear factor of activated T cells (NFAT) transcription factors are required for induction of T-cell cytokine production and effector function. Although it is known that activation via the T-cell antigen receptor (TCR) results in 2 critical steps, calcineurin-mediated NFAT1 dephosphorylation and NFAT2 up-regulation, the molecular mechanisms underlying each are poorly understood. Here we find that T cell p38, which is activated by an alternative pathway independent of the mitogen-activated protein (MAP) kinase cascade and with different substrate specificities, directly controls these events. First, alternatively (but not classically) activated p38 was required to induce the expression of the AP-1 component c-Fos, which was necessary for NFAT2 expression and cytokine production. Second, alternatively (but not classically) activated p38 phosphorylated NFAT1 on a heretofore unidentified site, S79, and in its absence NFAT1 was unable to interact with calcineurin or migrate to the nucleus. These results demonstrate that the acquisition of unique specificities by TCR-activated p38 orchestrates NFAT-dependent T-cell functions.

## Author summary

The p38 MAP kinase, which is required for a large number of important biological responses, is activated by an enzymatic cascade that results in its dual phosphorylation on p38^T180Y182^. T cells have evolved a unique pathway in which T-cell antigen receptor (TCR) ligation results in phosphorylation of p38^Y323^ (the alternative pathway). Why T cells acquired this pathway is the subject of conjecture. In this study, we examine the activation of 2 members of the nuclear factor of activated T cells (NFAT) family, which, when dephosphorylated by calcineurin, migrate from the cytoplasm to the nucleus. In T cells with the alternative pathway ablated by a single amino acid substitution (p38^Y323F^), NFAT1 remained in the cytoplasm after stimulation via the TCR. Studies identified NFAT1^S79^ as a target for alternatively (but not classically) activated p38, and phosphorylation of this residue was required for binding calcineurin and nuclear translocation. Furthermore, although classically activated p38 induced NFAT1 translocation in the absence of NFAT1^S79^ phosphorylation, unlike alternatively activated p38 it did not cause NFAT2 up-regulation. This paradox was resolved by the finding that only the latter induces c-Fos, which binds to the NFAT2 promoter and participates in its up-regulation. These T-cell-specific p38 activities provide a strong rationale for the acquisition of the alternative mechanism for activating p38.

## Introduction

Many activation events downstream of the T-cell antigen receptor (TCR) are up-regulated by the nuclear factor of activated T cells (NFAT) transcription factor family [1]. There are 5 NFAT family members: NFAT1 (also known as NFATc2 or NFATp), NFAT2 (NFATc1 or NFATc), NFAT3 (NFATc4), NFAT4 (NFATc3 or NFATx), and NFAT5 (tonicity-responsive enhancer binding protein [TonEBP]) [2]. T cells express NFAT1, NFAT2, and NFAT4, which are activated by TCR-mediated stimulation to induce proliferation and cytokine secretion [2]. Whereas expression of NFAT2 is inducible, NFAT1 and NFAT4 are constitutively expressed [2]. In resting cells, NFATs are highly phosphorylated by a number of serine/threonine kinases (e.g., casein kinase 1, glycogen synthase kinase 3, Jun N-terminal kinases, and p38), with as many as 21 phosphorylation sites having been identified in NFAT1 [3]. The phosphorylated species are retained in the cytosol (and thus inactive) by binding cytoplasmic proteins such as 14-3-3 [4]. Stimuli that increase cytosolic calcium activate the Ca^2+^-dependent serine-threonine phosphatase calcineurin, which dephosphorylates NFATs and allows their migration to the nucleus, where they induce gene transcription [2]. In T cells, stimulation via the TCR also induces NFAT2 expression [5]. NFATs often up-regulate gene transcription in cooperation with other transcription factors, most commonly AP-1, the combination being required for the expression of interleukin (IL)-2 [6], IL-3 [7], granulocyte-macrophage colony-stimulating factor (GM-CSF) [8], IL-4 [9], IL-5 [10], IL-13 [6], interferon gamma (IFN-γ) [11], Fas ligand (FasL) [12], CD25 [13], Cox2 [14], IL-8, and migration inhibitory factor 1 alpha (MIF-1α) [11]. Other reported partners include Sp1 and activating transcription factor 2 (ATF2)/Jun to induce expression of tumor necrosis factor alpha (TNF-α) [15] and myocyte enhancer factor-2 (MEF-2) to induce Nurr77 [16].

p38, a mitogen-activated protein kinase (MAPK), has important positive regulatory roles in a variety of cellular functions, including proliferation, proinflammatory cytokine production, and apoptosis [17]. In the classical MAPK cascade, stimuli such as environmental stress or proinflammatory cytokines activate a kinase cascade that results in the phosphorylation of p38 on Thr-180 and Tyr-182 (T180/Y182) in the activation loop [17]. In addition to this enzymatic cascade, T cells possess a unique mode of p38 activation that is downstream of the TCR. Stimulation via the TCR activates the protein tyrosine kinase zeta-associated protein (ZAP-70), which phosphorylates p38 on Tyr-323 (p38 pY323), leading to autophosphorylation on Thr-180 (the p38 alternative pathway) [18]. The adaptor protein hDlg1 has been implicated in coupling TCR signaling to alternative p38 activation, as its small interfering RNA (siRNA)-mediated knockdown reduces p38 activity [19]. The alternative p38 pathway is essential for the expression of several key T-cell effector functions, best shown in mice lacking the alternative pathway because of knock-in of p38α and p38β in which Tyr-323 was replaced with Phe (double knock-in [DKI] mice) [20]. TCR-activated cells from such animals produce low levels of proinflammatory cytokines such as TNF-α, IFN-γ, and IL-17A compared to wild-type (WT) cells. Importantly, the mice are resistant to the autoimmune disease models collagen-induced arthritis and experimental autoimmune encephalomyelitis (EAE) [20], have less aggressive Kras-induced inflammatory pancreatic cancer [21], and fail to mount an effective IL-17A-dependent immune response to *Citrobacter rodentium* infection [22].

In T cells, p38 activated via the MAPK cascade phosphorylates NFAT1 and NFAT2 on serine/threonine residues, resulting in cytosolic retention and inhibition [23]. It is well documented, however, that p38 is also a positive regulator of TCR-induced and NFAT-dependent cytokine production [24]. For instance, p38 is upstream of the up-regulation of NFAT2 upon TCR stimulation [5] and activates NFAT1 via phosphorylation of S54 in its transactivation domain [3,19]. In this study, we have addressed the molecular mechanisms by which p38 regulates NFAT1 and NFAT2. We found that the T-cell-specific p38 alternative pathway controls NFAT activation and dependent cytokine secretion by 2 mechanisms: (1) up-regulating c-Fos, which is required for induction of NFAT2 expression, and (2) phosphorylating NFAT1 S79 to allow its interaction with calcineurin and subsequent nuclear translocation.

## Results

### TCR-mediated up-regulation of NFAT2 requires p38 pY323-dependent induction of c-Fos

**Fig 1:**
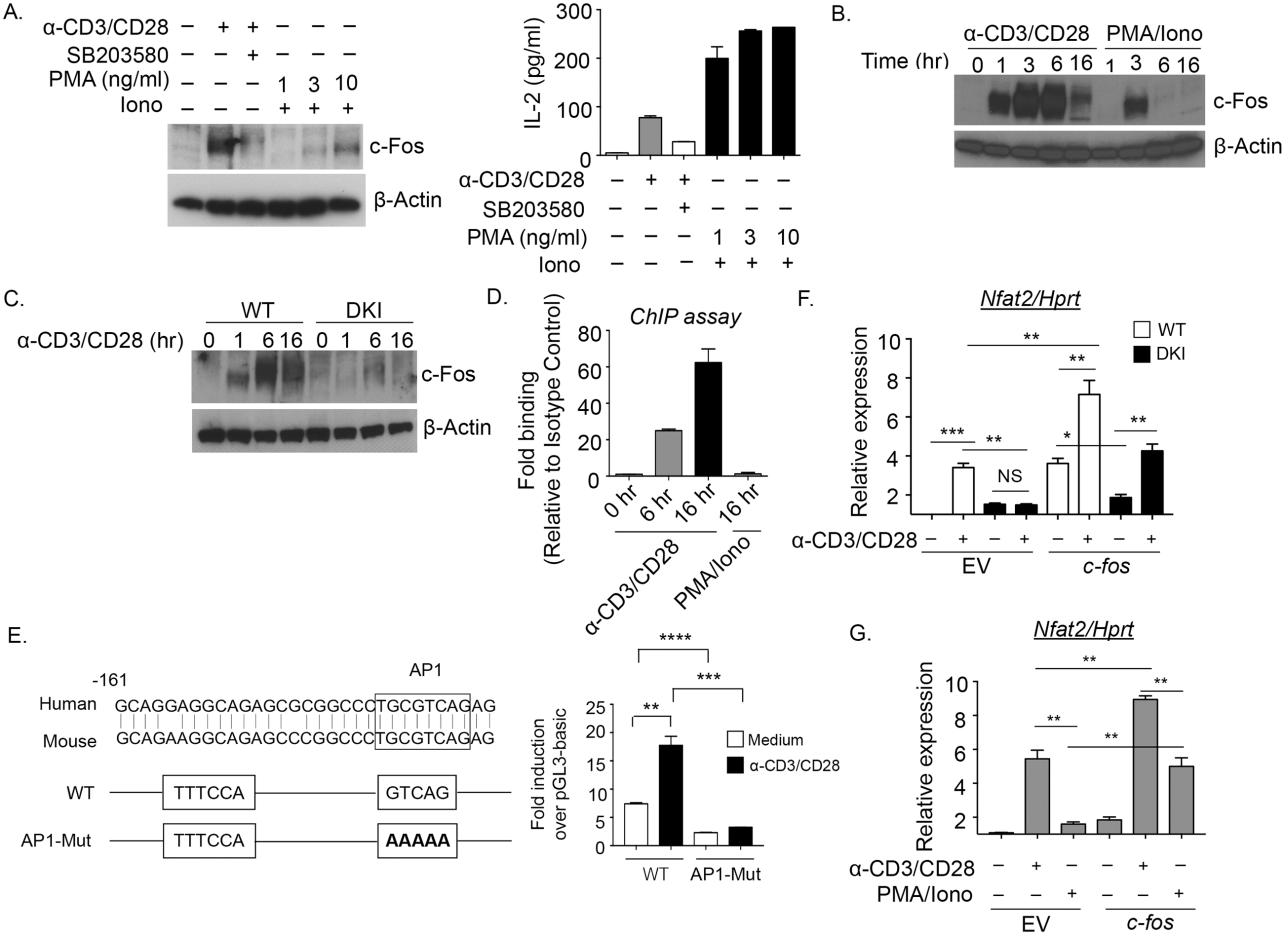
p38 Alternative activation is required for T-cell antigen receptor (TCR)-induced expression of NFAT2 via c-Fos. (A, left panel) Purified mouse T cells were stimulated with anti-CD3/CD28 in the presence or absence of SB203580 and phorbol myristate acetate (PMA) plus ionomycin for 16 hours and then immunoblotted. (A, right panel) Purified mouse T cells were stimulated as in panel A, and interleukin (IL)-2 was measured in the culture supernatant (S1 Data). (B) Purified T cells from wild-type (WT) mice were stimulated with anti-CD3/CD28 or PMA/ionomycin for the indicated times, and then the lysates were immunoblotted. (C) Purified T cells from WT or double knock-in (DKI) mice were stimulated with anti-CD3/CD28 for the indicated times, and then the lysates were immunoblotted. (D) Purified WT T cells were stimulated with anti-CD3/CD28 or PMA/ionomycin for the indicated times, and binding of c-Fos to the NFAT2 promoter was analyzed by chromatin immunoprecipitation (ChiP) (S1 Data). (E) pGL3-basic luciferase reporter construct containing the *nfat2* promoter consensus AP-1 binding site or its mutant (left panel) was transfected into Jurkat cells, which were stimulated overnight with anti-CD3/CD28, and then a luciferase assay was performed (right panel) (S1 Data). (F) Purified T cells from 2 WT or 2 DKI mice were infected with retrovirus carrying empty vector (EV) or *c-fos*. The T cells were pooled per group and stimulated with anti-CD3/CD28 for 24 hours, and the expression of *nfat2* mRNA was determined by quantitative real-time PCR. *nfat2* levels in WT EV-transduced and unstimulated samples were defined as 1 (S1 Data). (G) Purified murine T cells were infected and stimulated with anti-CD3/CD28 or PMA/ionomycin, and *nfat2* mRNA expression was quantitated by real-time PCR as in panel F (S1 Data). The results are representative of 3 independent experiments, and the bar graphs in the right panel show the mean ± SEM of all 3. *p < 0.05, **p <0.01, ***p < 0.001. NS, not significant.

Activation of p38 via the alternative pathway (but not the classical MAPK cascade) is required for up-regulation of NFAT2, its downstream target IRF4, and proinflammatory cytokine production [22]. In T cells, NFAT2 expression has been reported to be regulated by NFAT1 [25] and/or NFAT2 itself in association with the AP-1 transcription factor [26]. AP-1, a heterodimer of c-Fos and c-Jun, plays an important role along with NFATs in the induction of a number of T-cell products [26]. Because c-Fos is expressed only at low levels in resting T cells [27], we asked if its induction upon activation is dependent on p38. Little c-Fos was detected in primary resting T cells, but it was markedly induced after stimulation with anti-CD3/CD28 (Fig 1A, left lanes). The induction was blocked by the p38 catalytic inhibitor SB203580, suggesting a role for this kinase. Notably, activation of T cells with phorbol myristate acetate (PMA) plus ionomycin, which activates p38 via the classic MAPK cascade, was relatively ineffective at inducing c-Fos (Fig 1A, right lanes). Unlike anti-CD3/CD28 stimulation, PMA/ionomycin poorly induced c-Fos expression, which peaked at 3 hours but was not sustained at the later time points (Fig 1B). This was not due to the poor activation by PMA/ionomycin, because the combination induced robust IL-2 production (Fig 1A, right panel). To directly test if alternatively activated p38 was required, c-Fos expression was measured in primary T cells from WT or p38α and β DKI mice that lack the alternative pathway. In contrast to WT T cells, there was little if any c-Fos upregulation in T cells from DKI mice (Fig 1C). There was no difference in c-Jun expression between the WT and DKI T cells (S1 Fig). Therefore, alternatively activated p38 is necessary for c-Fos induction in cells activated via the TCR.

Given that up-regulation of both c-Fos and NFAT2 requires alternative p38 activation and that c-Fos is an immediate early gene, whereas NFAT2 induction is late [22], we asked if c-Fos regulates NFAT2 expression. Chromatin immunoprecipitation (ChIP) was performed on extracts from primary T cells using anti-c-Fos. In cells stimulated with anti-CD3/CD28, but not with PMA/ionomycin, there was clearly inducible binding of c-Fos to the NFAT2 promoter (Fig 1D). To determine if this binding was functionally important, the AP-1 binding site GTCAG in the *nfat2* promoter was mutated to AAAAA, inserted into a luciferase reporter plasmid, and transfected into the human Jurkat T leukemia cell line. Anti-CD3/CD28 induced luciferase production from a vector containing the WT *nfat2* promoter but had no effect when the AP-1 binding site was mutated (Fig 1E). To determine if p38-mediated c-Fos up-regulation was the only limiting factor in NFAT2 induction in DKI T cells, we infected primary mouse WT or DKI T cells with a retrovirus encoding *c-fos*. The expression vector included an internal ribosome entry site (IRES) upstream of a sequence encoding enhanced green fluorescent protein (eGFP) so that transfection efficiency could be monitored. Transfection efficiency was comparable for the empty vector (EV) control and the vector encoding *c-fos* (S2A Fig). Real-time quantitative reverse transcription PCR (qRT-PCR) revealed that anti-TCR-mediated activation of WT T cells transduced with EV up-regulated *nfat2* mRNA, whereas there was no response in EV-transduced DKI T cells, consistent with our previous report (Fig 1F) [22]. Importantly, DKI T cells in which *c-fos* was introduced up-regulated *nfat2* in response to TCR signaling. Interestingly, the baseline *nfat2* levels in *cfos*-transduced cells was elevated, probably reflecting the fact that the cells had recently been activated to facilitate retroviral transduction. Similar experiments were performed with PMA/ionomycin-stimulated WT cells, in which NFAT2 is poorly induced [22]. Enforced expression of *c-fos* allowed T cells to up-regulate *nfat2* when stimulated with PMA/ionomycin (Fig 1G and S2B Fig). Therefore, p38 activated via the TCR uniquely up-regulates c-Fos expression, which is essential for downstream induction of NFAT2.

### TCR-induced nuclear translocation of NFAT1 requires the p38 alternative pathway

**Fig 2:**
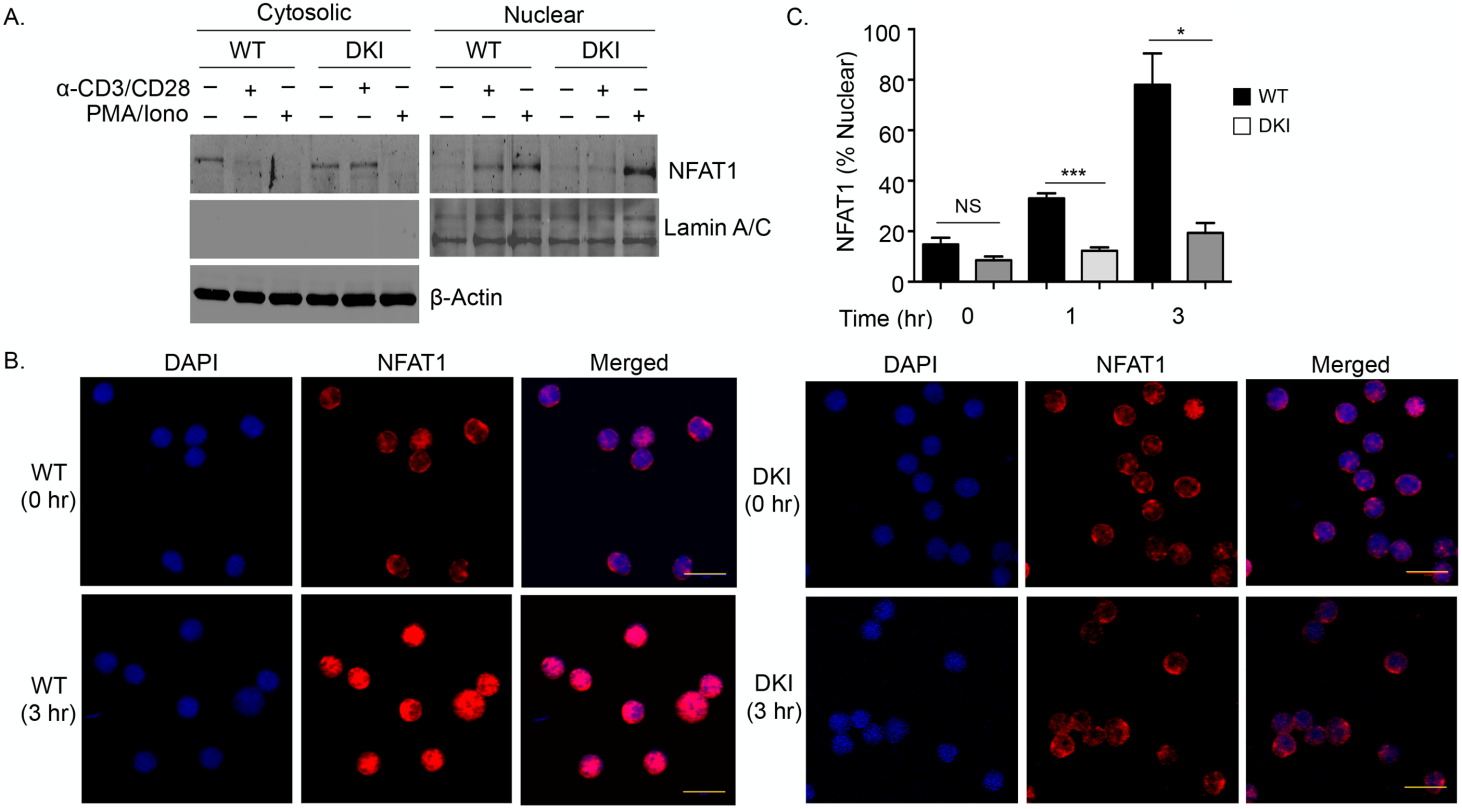
The p38 alternative pathway is required for NFAT1 nuclear migration. (A) Purified T cells from wild-type (WT) or double knock-in (DKI) mice were stimulated with anti-CD3/CD28 or phorbol myristate acetate (PMA)/ionomycin for 3 hours, and NFAT1 levels in the nuclear and cytosolic fractions were determined by immunoblotting. (B) Purified T cells from WT or DKI mice were stimulated as in panel A, and NFAT1 (red) localization was visualized by confocal microscopy. DAPI (blue) was used to stain the nucleus. Scale bar = 10 μM. (C) Quantification of the percentage of cells with nuclear NFAT1 per high power field (HPF). Error bars are the mean ± SEM (S2 Data). *p < 0.05, ***p < 0.001. NS, not significant.

Unlike NFAT2, NFAT1 is constitutively expressed, and activation-induced increases in cytosolic Ca^2+^ cause its dephosphorylation by calcineurin and nuclear migration [2]. Because NFAT1 activity was increased in T cells in which hDlg1 was overexpressed and decreased when p38 was inhibited [19], we directly asked if p38 is required at this early step. Purified T cells from WT or DKI mice were cultured in medium alone or stimulated with anti-CD3/CD28 for 3 hours, and the cytoplasmic versus nuclear distribution of NFAT1 was determined. As expected, in resting WT T cells, NFAT1 was entirely cytoplasmic, but after anti-TCR-mediated activation, it was found in the nucleus (Fig 2A). In contrast, in T cells from DKI mice that lack the alternative p38 activation pathway NFAT1 did not translocate to the nuclear fraction after activation. This was specific to TCR-mediated signaling, because NFAT1 translocated to the nucleus in both WT and DKI T cells stimulated with PMA plus ionomycin. This was confirmed by confocal microscopy. Whereas in resting T cells of both genotypes NFAT1 was largely excluded from the nucleus, after anti-CD3/CD28 stimulation NFAT1 translocated to the nucleus only in WT T cells, remaining in the cytosol of DKI T cells (Fig 2B and 2C). Therefore, the p38 alternative activation pathway is required for NFAT1 activation after TCR-mediated stimulation.

### ZAP-70-activated, but not mitogen-activated protein kinase kinase 6 (MKK6)-activated, p38 phosphorylates NFAT1 on S79

**Fig 3:**
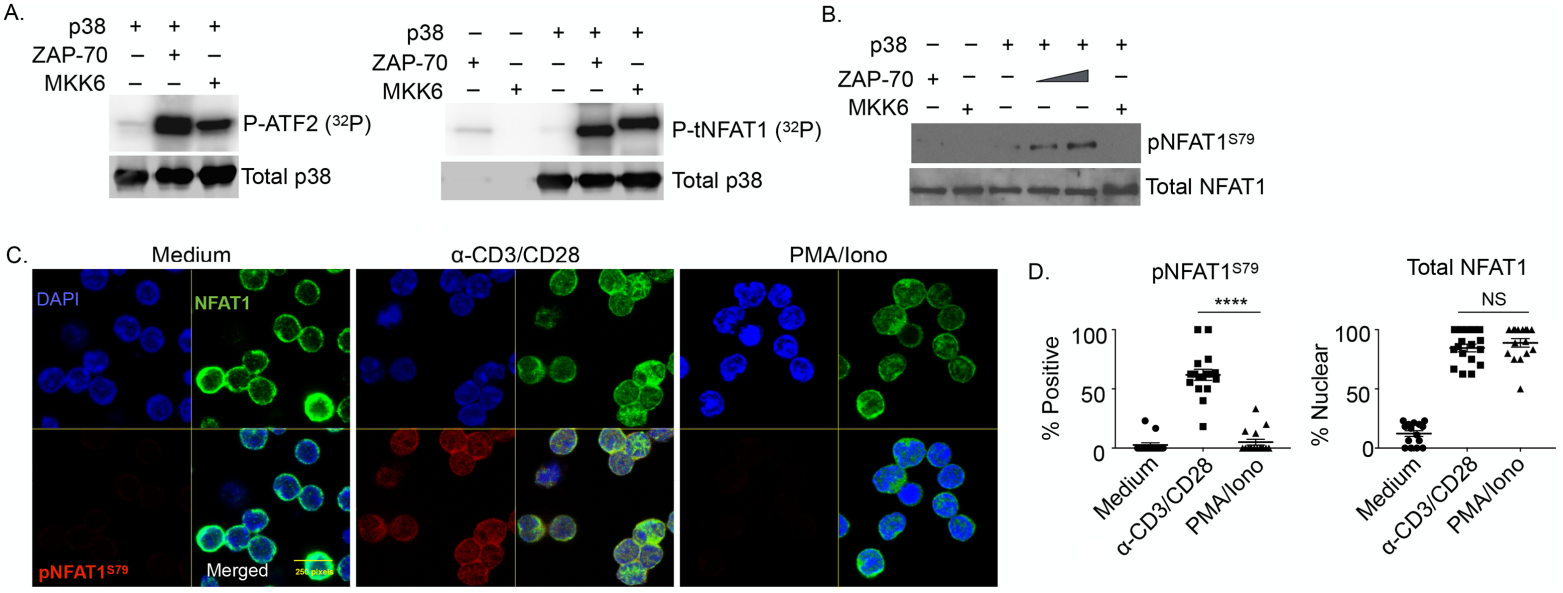
Alternatively activated p38 uniquely phosphorylates NFAT1 on S79. (A) In vitro kinase assay in which recombinant mouse p38α was incubated with active human zeta-associated protein (ZAP-70), mitogen-activated protein kinase kinase 6 (MKK6), or buffer alone. After 1 hour, recombinant ATF2 (left panel) or tNFAT1 (right panel) and 10 μCi [^32^P]ATP were added for 30 minutes before separation on SDS-PAGE and PhosphorImager analysis. The results are representative of 3 independent experiments. (B) Recombinant mouse p38α was incubated with active human ZAP-70, MKK6, or buffer alone in in vitro kinase buffer. After 1 hour, recombinant tNFAT1 was added and incubated for an additional hour before separation on SDS-PAGE and immunoblotting with antibodies specific for pNFAT1^S79^. (C) Freshly purified wild-type (WT) T cells were stimulated with anti-CD3/CD28, phorbol myristate acetate (PMA)/ionomycin, or buffer alone for 15 minutes. Cells were stained for total NFAT1 (green) or pNFAT1^S79^ (red) and imaged by confocal microscopy. Scale bar = 250 pixels. (D) Quantification of the percent of cells positive for NFAT1^pS79^ (left panel) and percent of cells with nuclear NFAT1 (right panel) per high power field (HPF) in the experiment shown in panel C. Each dot represents an individual HPF (S3 Data). ****p < 0.0001. NS, not significant.

NFAT1 is a target of classically activated p38, but these phosphorylation events are thought to be responsible for cytosolic retention [23]. The finding that p38 activity was actually required for NFAT1 translocation downstream of the TCR, therefore, raised the possibility that the classic and alternative p38 pathways lead to differential NFAT1 phosphorylation. To determine if NFAT1 is a substrate of alternatively activated p38 and, if so, whether the sites of phosphorylation differ from those of MKK-activated p38, an in vitro kinase assay was performed using recombinant p38 that had been activated by ZAP-70 (alternative pathway) or MKK6 (MAPK cascade). As substrate we used an NFAT1 fragment containing the first 350 amino acids (tNFAT1), in which the bulk of the known phosphorylation sites have been identified [3], or ATF2 as a positive control. Both ZAP-70- and MKK6-phosphorylated p38 phosphorylated ATF2, the former actually having more activity (Fig 3A, left panel). They also phosphorylated tNFAT1, with MKK6-activated p38 presumably phosphorylating more sites as manifested by slower migration in SDS-PAGE (Fig 3A, right panel). This was confirmed by mass spectrometry. Whereas 5 phosphorylated serine residues (54, 110, 136, 150, and 223, 4 of which have been reported in a resting T-cell clone [3]) were identified in tNFAT1 incubated with classically activated p38, only one phosphorylation site was detected in tNFAT1 incubated with ZAP-70 activated p38, serine 79 (S79) (S3 Fig; S1 Table). S79 is in the transactivation domain of NFAT1 and to our knowledge has not been previously identified as a phospho-acceptor site.

We generated a rabbit antiserum against an NFAT1 peptide containing phosphorylated Ser-79 (pNFAT1^S79^). The affinity-purified antibodies were highly selective for the phosphorylated species, as measured by ELISA (S4 Fig). In vitro kinase assays with recombinant p38 confirmed that ZAP-70-activated (but not MKK6-activated) p38 phosphorylated NFAT1 on Ser-79 (Fig 3B). The anti-pNFAT1^S79^ antibody was used in confocal imaging of T cells that were stimulated with either anti-CD3/CD28 or PMA/ionomycin. NFAT1 (green) was non-nuclear (blue) in unstimulated cells, and no pNFAT1^S79^ (red) was detected. NFAT1 migrated to the nucleus upon both anti-CD3/CD28 or PMA/ionomycin stimulation, but Ser-79 phosphorylation was only detected in the nuclei of T cells stimulated with anti-CD3/CD28 (Fig 3C). Analysis of cells from multiple experiments found that approximately 60% of the T cells activated via the TCR had nuclear NFAT1, all of which were positive for pNFAT1^S79^ (Fig 3D, left panel). In contrast, after PMA/ionomycin stimulation, NFAT1 was detected in the nucleus of approximately 80% of the T cells, but no pNFAT1^S79^ was found (Fig 3D, right panel). Thus, TCR signaling (but not the MAPK cascade) results in phosphorylation of NFAT1 on Ser-79.

### Phosphorylation of NFAT1^S79^ is required for TCR-mediated IL-2 and TNF-α production

**Fig 4:**
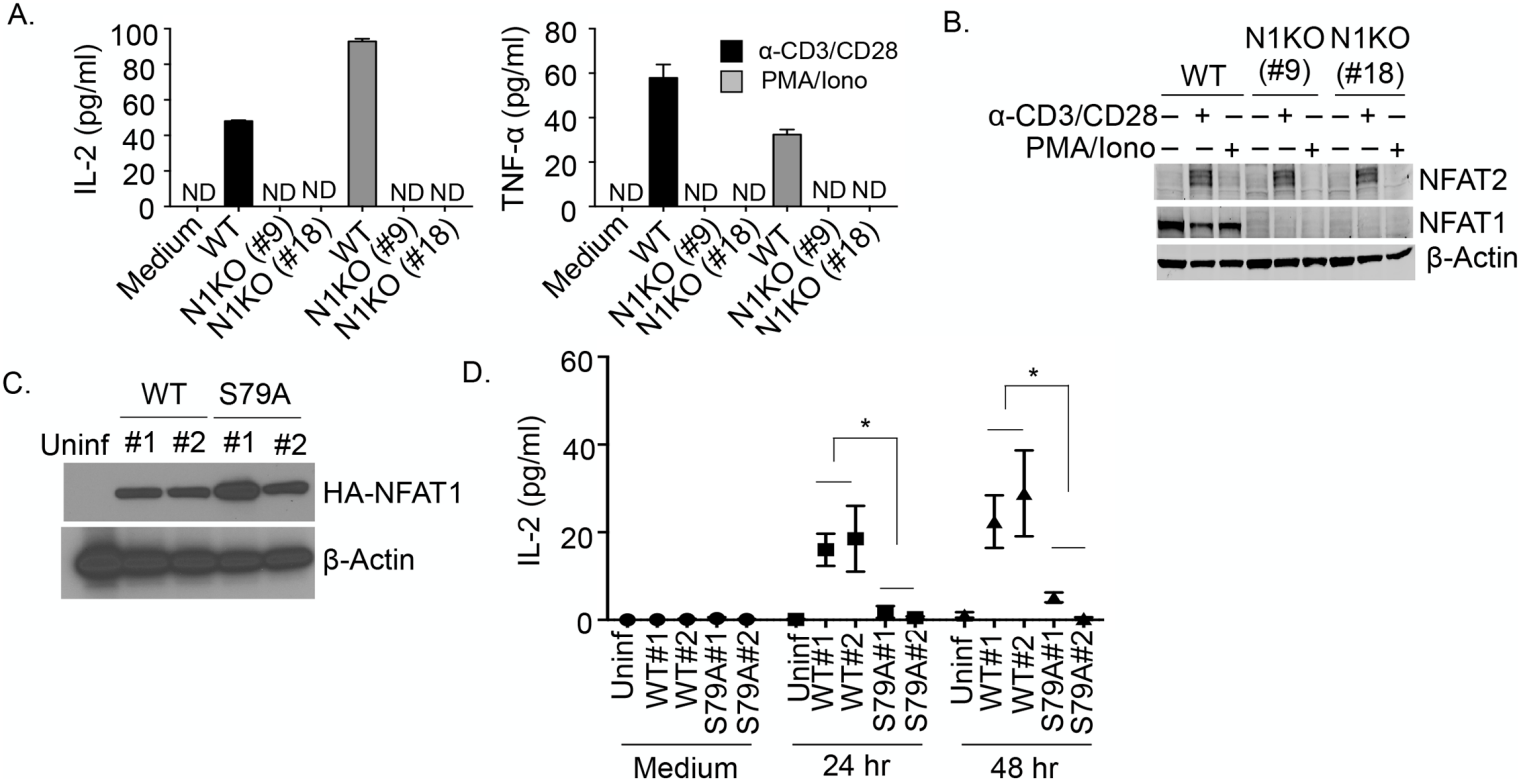
Role of NFAT1 and NFAT1^S79^ in NFAT2 and cytokine expression. (A) Interleukin (IL)-2 and tumor necrosis factor alpha (TNF-α) production in supernatants of wild-type (WT) or N1KO Jurkat clones stimulated with anti-CD3/CD28, phorbol myristate acetate (PMA)/ionomycin, or medium alone for 20 hours. The results represent the mean of 3 independent experiments ± SEM (S4 Data). (B) WT or N1KO Jurkat clones were stimulated with anti-CD3/CD28, PMA/ionomycin, or medium alone for 48 hours, and NFAT2 expression was determined by immunoblotting. (C) The N1KO Jurkat clone was infected with retrovirus encoding HA-NFAT or HA-NFAT1^S79A^, followed by single cell sorting of green fluorescent protein-positive (GFP^+^) cells. Quantitation of transduced gene product expression in 2 independent clones from each transduction was determined by immunoblotting with anti-HA. (D) Quantitation of IL-2 in the supernatants of HA-NFAT1 or HA-NFAT1^S79A^ Jurkat clones stimulated with anti-CD3/CD28, PMA/ionomycin, or medium alone. The results represent the mean of 3 independent experiments ± SEM (S4 Data).

To determine the biological relevance of NFAT1^S79^ phosphorylation, NFAT1 was targeted using CRISPR-Cas9 technology in Jurkat cells (N1KO cells). WT Jurkat and 2 independent N1KO clones (#9 and #18) had similar levels of TCR-β and CD3 expression (S5 Fig). In contrast to WT Jurkat cells, the N1KO cells stimulated with anti-CD3/CD28 or PMA/ionomycin failed to produce IL-2 or TNF-α (Fig 4A). Interestingly, despite a report that NFAT1 is upstream of NFAT2 in murine primary T cells stimulated with PMA/ionomycin [25], in the absence of NFAT1, anti-TCR activation of the N1KO clones induced NFAT2 expression to the same level as in WT Jurkat (Fig 4B). Therefore, at least in Jurkat cells, NFAT1 is not required for NFAT2 expression. As previously shown, stimulation with PMA/ionomycin induced little if any NFAT2 (Fig 4B) [22]. The N1KO cells (clone #9) were retrovirally transduced with vectors encoding HA-NFAT1 or NFAT1^S79A^, and 2 independent clones of each that had similar levels of HA-NFAT1 were selected for study (Fig 4C). Importantly, introduction of NFAT1 but not NFAT^S79A^ restored activation-induced IL-2 secretion (Fig 4D). Therefore, T-cell p38-mediated phosphorylation of NFAT1^S79^ is essential for NFAT1 nuclear translocation and downstream gene transcription in response to TCR signaling.

### TCR-induced phosphorylation of NFAT1^S79^ is required for calcineurin binding and nuclear translocation

**Fig 5:**
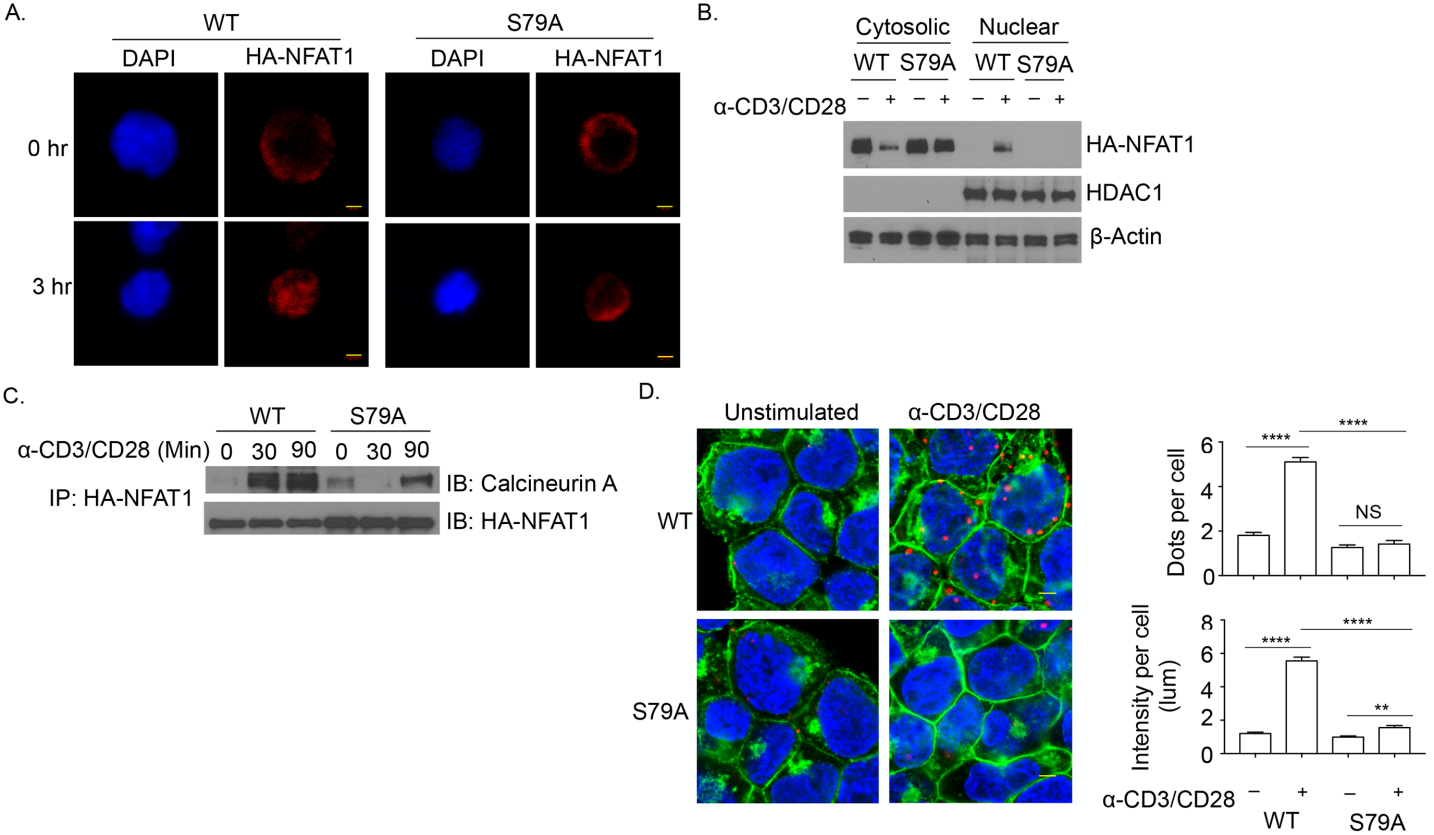
Phosphorylation of NFAT1^S79A^ is required for nuclear migration upon T-cell antigen receptor (TCR) stimulation. (A) Purified T cells from wild-type (WT) mice were infected with retrovirus encoding HA-NFAT1 or HA-NFAT1^S79A^. The cells were stimulated with anti-CD3/CD28 for 1 hour and examined for NFAT1 (red) localization by confocal microscopy. DAPI was used to stain the nucleus. Scale bar = 10 μM. (B) Purified primary T cells from WT mice were stimulated and infected as in panel A, and NFAT1 levels in the cytosolic and nuclear fractions were assessed by immunoblotting. (C) Stable Jurkat cell lines expressing HA-NFAT1 or HA-NFAT1^S79A^ were stimulated with anti-CD3/CD28 for 1 hour, and the lysates were immunoprecipitated (IP) with anti-HA and immunoblotted (IB) for calcineurin A and HA. (D) Confocal images of in situ proximity ligation assay (PLA) of stable Jurkat cell clones expressing HA-NFAT or HA-NFAT1^S79A^ that had been stimulated with anti-CD3/CD28 for 15 minutes. Alexa Fluor 488 (green)-conjugated wheat germ agglutinin (WGA) was used to stain plasma membrane. Scale bar = 100 pixels (left panel). Quantification of the average dots and intensity per cell (WT-Uns [*n* = 166], anti-CD3/CD28 [*n* = 132]; S79A-Uns [*n* = 162], and anti-CD3/CD28 [*n* = 132]) (right panel) (S5 Data). **p < 0.01, ****p < 0.0001. NS, not significant.

To investigate the functional significance of pNFAT1^S79^, NFAT1-deficient Jurkat cells were infected with retrovirus encoding HA-NFAT1 or HA-NFAT1^S79A^. The cells were stimulated with anti-CD3/CD28 or PMA/ionomycin, and the cellular localization of the HA-tagged proteins was determined by confocal microscopy. Infection efficiency was similar between the HA-NFAT1- and HA-NFAT1^S79A^-expressing cells (S6A Fig). Whereas the HA-tagged proteins were located in the cytosol of unstimulated cells, activation with anti-CD3/CD28 induced nuclear translocation of HA-NFAT1, but not of HA-NFAT1^S79A^ (S6B Fig). This was confirmed by immunoblotting for HA in the cytoplasmic and nuclear fractions of anti-CD3/CD28-stimulated cells (S6C Fig). Anti-CD3/CD28 caused the nuclear translocation of NFAT1, but not of NFAT1^S79A^. In response to PMA/ionomycin, there was only a modest impairment of NFAT1^S79A^, but to a much less extent than NFAT1. Similar results were obtained with primary murine T cells, which were retrovirally transduced with HA-NFAT1 or HA-NFAT1^S79A^ (S6D Fig). As observed in Jurkat cells, HA-NFAT1 but not HA-NFAT1^S79A^ migrated to the nucleus upon stimulation with anti-CD3/CD28 (Fig 5A and 5B).

Given that calcineurin-mediated dephosphorylation is required for NFAT1 nuclear translocation, we characterized the interaction between these proteins. Stable Jurkat cell lines expressing HA-NFAT1 or HA-NFAT1^S79A^ were stimulated with anti-CD3/CD28, the lysates were immunoprecipitated with anti-HA, and the immunoprecipitated material was immunoblotted for the catalytic subunit, calcineurin A. Although total cell lysates had similar amounts of HA-NFAT1 and calcineurin (S6E Fig), there was much more calcineurin coimmunoprecipitated with HA-NFAT1 than HA-NFAT1^S79A^ (Fig 5C). One way to assess molecular interactions in situ is a proximity ligation assay (PLA), in which protein-protein interactions (<40 nm) can be visualized as individual dots by microscopy. PLA revealed a robust interaction of NFAT1 and calcineurin (red) in anti-TCR-activated cells expressing HA-NFAT1, but not in HA-NFAT1^S79A^ (Fig 5D, left panel). Quantitative analysis showed that both the number and the intensity of dots were much greater in anti-CD3/CD28-stimulated HA-NFAT1-expressing cells than in HA-NFAT1^S79A^-expressing cells (Fig 5D, right panel). These results indicated that in TCR-stimulated cells alternatively activated p38 phosphorylates NFAT^S79^, which is necessary for binding to calcineurin and subsequent nuclear migration.

## Discussion

Studies with gene-targeted mice have shown that NFAT family members have both unique and overlapping functions in many tissues. For example, germ line deletion of NFAT2 results in embryonic lethality due to a failure of cardiac valve formation [28], and its disruption in embryonic stem cells causes a substantial defect in osteoclast differentiation [29]. NFAT1 knockout mice survive but have a substantial reduction in mast cell cytokine production and T-cell-produced TNF-α, but not IL-4 [30,31]. Functional redundancy between some family members was inferred from the mild phenotypes found in some single-gene disruptions [32], and a number of studies have been carried out in animals in which multiple family members were deleted For example, deletion of NFAT3 and NFAT4 resulted in excessive and disorganized neural tubes in the embryo [33], and deletion of NFAT1, NFAT3, and NFAT4 impaired axonal outgrowth in the central and peripheral nervous systems [34]. Although T cells express NFAT4, NFAT1 and NFAT2 appear to play the predominant role in T-cell activation and function [35]. Deletion of both NFAT1 and NFAT2 prevented cytokine production by T cells stimulated with anti-CD3/CD28 [11], whereas the introduction of constitutively active forms of both increased the expression of many cytokines in response to anti-CD3/CD28 or PMA/ionomycin stimulation [36,37]. Nonredundant functions of NFAT1 and NFAT2 in T cells were revealed in studies of mice deficient in one or both [38]. When immunized with myelin oligodendrocyte glycoprotein (MOG) to induce EAE, NFAT1-deficient mice produced less TNF-α, whereas NFAT2-deficient mice produced less IL-17A and IL-10. Consistent with this, overexpression of NFAT1 (but not of NFAT2) induced TNF-α production in human T cells stimulated via the TCR [39].

Although NFATs have been extensively investigated, much of the mechanistic information about their regulation has come from studies using Ca^2+^ ionophores rather than physiologic stimuli. Whereas NFAT1 expression is constitutive, NFAT2 is expressed at low levels in resting T cells and induced by stimulation via the TCR. We have previously shown that the alternative p38 pathway is required for up-regulation of NFAT2, because it is poorly induced in either WT T cells activated with PMA/ionomycin or, notably, in DKI T cells stimulated via the TCR [22]. This was in agreement with the finding that siRNA-mediated knockdown of the p38-binding scaffold protein Dlgh1 inhibited TCR-mediated p38 activation and downstream *Nfat2* mRNA induction [19]. There is relatively little known about the transcription factors that mediate TCR-signaled *nfat2* up-regulation. The role of NFAT1 is controversial. Although NFAT2 expression was higher in the nucleus of *nfat1*^-/-^ CD4^+^ T cells [38], NFAT2 reporter activity was decreased in NFAT1-deficient T cells stimulated with PMA/ionomycin [25]. NFAT2 may itself participate in its own up-regulation via a positive feedback loop [40], which leaves open the identity of the initiating factors. One clue suggesting the possibility that T-cell c-Fos may be involved comes from bone marrow-derived macrophages, in which receptor activator of nuclear factor κB ligand (RANKL)-induced osteoclast differentiation was impaired in *c-fos*^*-/-*^ cells because of lack of NFAT2 expression [29]. This, and the fact that AP-1 is often required as an NFAT1 cofactor [10,41,42], led us to examine *c-fos* expression downstream of TCR stimulation. c-Fos, like NFAT2, was up-regulated robustly, in marked contrast to the poor and transient induction in response to PMA/ionomycin. Analysis by ChIP showed that c-Fos bound the *nfat2* promoter, and enforced expression of c-Fos allowed NFAT2 up-regulation in PMA/ionomycin-stimulated WT T cells and TCR-stimulated DKI T cells, demonstrating that c-Fos is a limiting factor for NFAT2 expression. Although PMA/ionomycin induced c-Fos expression, it was not sustained, consistent with the ChIP assay performed 16 hours after stimulation. What is downstream of alternative p38 signaling that up-regulates c-Fos expression is not known. One possibility is c-Fos itself, as it has been suggested that it can participate in its own up-regulation. In one example in National Institutes of Health (NIH) 3T3 cells (mouse fibroblast cell line), extracellular signal-regulated kinase (ERK)-mediated phosphorylation of c-Fos on serine/threonine residues preceding prolines recruits Pin1, a prolyl isomerase, resulting in further induction of *c-fos* in a feed-forward manner [43]. It is possible that p38 (also a proline-directed kinase) phosphorylates c-Fos on residues involved in Pin1 recruitment and c-Fos induction. Further studies will be required to elucidate the mechanism.

NFAT1 has been reported to have at least 21 serine phosphorylation sites, 13 of which are in the regulatory domain and are conserved among NFAT family members [3]. Although investigation of activation of NFATs has largely focused on their dephosphorylation, one inducible phosphorylation site in the NFAT1 transactivation domain, S54, has been described [3]. In that report, it was found that inducible phosphorylation of S54 was critical for its transcriptional activity in PMA/ionomycin-stimulated Jurkat cells [3]. We also found that MKK-phosphorylated p38 (classical pathway) caused NFAT1 S54 phosphorylation, along with 4 other sites critical for cytosolic retention. In contrast to MKK6-activated p38, ZAP-70-activated p38 phosphorylated NFAT1 at a single residue in the transactivation domain, S79, which we found was necessary for nuclear translocation. Nuclear shuttling of NFATs is largely regulated by calcineurin-mediated dephosphorylation, which exposes nuclear localization signal (NLS) regions [44], but how calcineurin targets NFATs in response to elevations in intracellular Ca^2+^ is not well understood. Here we have identified a new phosphorylation site, S79, that is necessary for calcineurin-NFAT1 association in TCR-signaled T cells. Both coimmunoprecipitation assays and PLAs demonstrated that phosphorylation of this site, which is near the NFAT1 calcineurin-binding sequence PxIxIT (residues 111–116), promotes NFAT1-calcineurin interactions. Whether this is a direct effect on NFAT-1-calcineurin binding or is indirect via, for example, enhanced phosphorylation of other residues or dissociation of an inactive cytoplasmic protein complex containing NFAT1 remains to be determined. Although we observed NFAT1 nuclear translocation in response to PMA/ionomycin, we were unable to detect phosphorylation of NFAT1^S79^. In this case, it may be that phosphorylation of NFAT1^S54^ is the functional equivalent. The identification of a mechanism for regulated access of calcineurin to its substrate NFAT1 identifies a new rate-limiting step in the propagation of signals from the TCR to the nucleus and eventual induction of effector cytokines.

MAPKs are proline-directed serine/threonine (S/T) kinases, meaning that the targeted S/T residues are followed by a proline (+1). Whereas ERK and c-Jun N-terminal kinase (JNK) have been shown to be strictly proline guided, p38 is more promiscuous [45]. Unlike ERK and JNK, in in vitro kinase assays, MKK6-activated p38 phosphorylated microtubule-associated Tau protein on residues in which proline was at the +1 position or the +3 position. Interestingly, among the latter was Tau residue Ser-185 in the sequence pSGEPPKS, which is quite similar to the region following NFAT1 Ser-79, pSGEPPGR. Note that in our case p38 was activated by ZAP-70, not MKK6, so this specificity is not inherent in the mode of activation but reflects a laxity in the consensus sequence not shared by other members of the MAPK family.

There is a longstanding paradox in T cells that activation of p38 is upstream of both NFAT1 inhibition (by phosphorylating residues that cause cytoplasmic retention) and the production of NFAT1-dependent cytokines [23]. The findings in the present report resolve this paradox by demonstrating that the alternative p38 pathway, which is the physiologic pathway downstream of TCR signaling, and the classic pathway have very different effects on NFAT1 and NFAT2 activation because of different substrate specificities. The widely studied stress-induced classic p38 pathway leads to phosphorylation of NFAT1 on inhibitory residues, preventing its nuclear migration, and fails to induce c-Fos and thus NFAT2. In contrast, alternatively activated p38 phosphorylates NFAT1 on S79, which promotes recruitment of calcineurin, dephosphorylation of inhibitory sites, and nuclear migration. Moreover, alternatively activated p38 signals for c-Fos up-regulation, which is required for NFAT2 induction. We have previously shown that differences in the substrate specificity of classically activated versus alternatively activated p38 result in antagonism of TCR-induced cytokine production and proliferation [22]. In that case, the latter induced NFAT2 expression, but the former blocked its function by directly phosphorylating S/T residues that prevented nuclear translocation. This report demonstrates the converse, in which the alternatively activated p38 has a specificity not shared by the classically activated form. T cell p38 is therefore a master regulator of NFATs, controlling the production of effector cytokines essential in adaptive immunity.

## Materials and methods

### Mice

Mice expressing p38αβ^Y323F^ (DKI mice) [20] were crossed onto C57BL/6 (B6) for at least 12 generations.

### Ethics statement

Mice were maintained in the National Cancer Institute pathogen-free animal facility, and all animal experiments were performed under an NCI ACUC-approved animal study protocol (LICB-008), which follows AAALAC guidelines.

### Reagents

Antibodies against mouse CD3 (145-2C11, BD Biosciences), CD28 (37.51, BD Biosciences), p38α (5F11; Cell Signaling), c-Fos for immunoblotting (sc-52 FITC; Santa Cruz Biotechnology), and c-Fos for immunoprecipitation (sc-52 X; Santa Cruz Biotechnology) were used. Antibodies against human CD3 (OKT3; eBioscience), CD28 (CD28.2; eBioscience), and NFAT1 (BD Biosciences) were used. Anti-β-actin (Sigma, A5441) and anti-HDAC1 (Cell Signaling Technology, 10E2) detect both human and mouse proteins. PMA and ionomycin were purchased from Sigma-Aldrich. Recombinant active human ZAP-70 was purchased from R&D System (NM_001079). [γ-^32^P]ATP was purchased from Perkin Elmer. Donkey anti-rabbit IgG-HRP (GE Healthcare, NA934V) was used as a secondary antibody for ELISA.

### Cells

Jurkat cells were purchased from ATCC (Clone E6-1). Primary T cells were purified from the spleens of 6–12-week-old B6 or DKI mice using negative-selection T-cell purification columns (CL101) from Cedarlane. Purified T cells and Jurkat cells were cultured in RPMI 1640 supplemented with 10% fetal bovine serum (Invitrogen), 2 mM glutamine, 50 μM β-mercaptoethanol, and 100 μM gentamicin. Plat E and Plat GP cells were cultured in DMEM supplemented with 10% fetal bovine serum (Invitrogen), 2 mM glutamine, 50 μM β-mercaptoethanol, and 100 μM gentamicin.

### Mass spectrometry

To identify sites of phosphorylation on tNFAT1, in vitro kinase reactions were separated by SDS-PAGE, and the tNFAT1 band was excised and subjected to in-gel trypsin digestion (Shevchenko PMID: 17406544); the resultant peptides were extracted and lyophilized to dry. Phosphopeptides were enriched using TiO_2_ magnetic sepharose (GE Healthcare) following manufacturer’s protocols. The flow-through and eluted peptides were desalted by C18 ZipTip (Millipore) before mass spectrometry analysis on an Orbitrap Fusion mass spectrometer (Thermo). The raw data were analyzed using Proteome Discoverer (Thermo).

### Production of antibodies to phospho-S79-NFAT1

C-terminal cysteine-containing peptides of NFAT1 corresponding to residues 74–85, which are conserved between human and mouse, without or with phosphorylated Ser-79 [Ac-PLASLpSGEPPGRC], were created by solid-phase peptide synthesis utilizing 9-fluorenylmethoxycarbonyl (Fmoc)/tert-butyl chemistry. The peptides were characterized by matrix-associated laser desorption ionization time-of-flight mass spectrometry (MALDI microMX, Waters), and purity (>95%) was confirmed with RP-HPLC. To conjugate with keyhole limpet hemocyanin (KLH), 6 mg of the phosphorylated peptide was dissolved in 120 μl PBS and mixed with 350 μl of IMA-KLH (10 mg/ml in H_2_O) (Thermo Fisher Scientific) and stirred at room temperature for 2 hours, followed by dialysis against PBS overnight. Antisera were raised by immunizing rabbits with the KLH-coupled phosphorylated peptide in Complete Freund’s Adjuvant (Pocono Rabbit Farm & Laboratory). Phospho-specific antibodies were isolated by affinity purification.

### Preparation of cytosolic and nuclear fractions

Cytosolic and nuclear fractions were collected using NE-PER nuclear and cytoplasmic extraction reagents (Thermo Scientific).

### ChIP assay

ChIP was performed using the Magna ChIP G kit (Millipore) with minor modifications according to the protocol published elsewhere [46]. Briefly, primary T cells were unstimulated or activated with anti-CD3/CD28 or PMA/ionomycin, fixed with 1% formaldehyde, and sonicated, and immunoprecipitation was performed with rabbit IgG control or rabbit anti-c-Fos antibody [47]. The coimmunoprecipitated DNA served as a template for subsequent PCR with primers encompassing the NFAT2 promoter: Fwd: 5′-TGA TGT CAC TGA AGG GAG GG-3′ and Rev: 5′-GGA GCC TCT CGG TCT CAC TCT G-3′). Quantitative real-time PCR consisted of an initial incubation for 1 minute at 95 °C followed by 30 seconds at 95 °C, 30 seconds at 52 °C, and 2 minutes at 72 °C. The reaction was subjected to 40 thermal cycles.

### NFAT1 knockout in Jurkat cells using CRISPR-Cas9

A CRISPR lentiviral vector (plentiCRISPRv2#52962, Addgene) carrying a Cas9 and puromycin resistance gene was used to clone guide RNA. The guide RNA sequences targeting NFAT1 were chosen from http://crispr.mit.edu. Oligonucleotides were designed for cloning into the vector as described [48,49]. The final guide sequences selected for NFAT1 were as follows: gRNA1: 5′-CACCGATCCGGCTCTCCGAATCGGC-3′; gRNA2: 5′-CACCGGACGGAGTGATCTCGATCCG-3′. The gRNAs were cloned into a BsmB1 site and subsequently sequenced using U-6 primer (5′-GAGGGCCTATTTCCCATGATT- 3′). To produce virus for infection, HEK 293T cells were transfected with 5 μg of gRNA1, 5 μg of gRNA2, 8 μg of packaging plasmid psPAX2 (Plasmid#12260, Addgene), and 2 μg of envelope-expressing plasmid pMD2.G (Plasmid#12259, Addgene) using Lipofectamine 2000 (Thermo Fisher). After overnight incubation, the old growth medium was replaced with 10 ml of fresh warm medium. Virus was harvested 48 hours post transfection by passing the supernatant through 0.45 μM filters (Millipore). Jurkat cells were infected with the virus, and 72 hours after infection, selection was performed using RPMI medium containing 2 μg/ml puromycin, which was replaced every 48 hours. After 2 weeks, single cells were sorted by BD FACSAria Fusion using live cell gating. Putative NFAT1 KO clones were screened for lack of NFAT1 expression by immunoblotting.

### Retroviral transduction

Purified mouse primary T cells were stimulated with plate-bound anti-CD3 (2 μg/ml) and anti-CD28 (2 μg/ml) for 24 hours and infected with retrovirus containing genes encoding HA-NFAT1, HA-NFAT1^S79A^, c-Fos, or EV. Cells were further stimulated with anti-CD3/CD28 for 48 hours followed by rest in medium alone for 24 hours before being stimulated in assays. WT Jurkat cells were infected with retrovirus carrying HA-NFAT or HA-NFAT1^S79A^ and cultured in complete medium for 48 hours before analysis. NFAT1 KO Jurkat cells were infected with retrovirus carrying HA-NFAT1 or HA-NFAT1^S79A^ followed by single-cell sorting for cells expressing GFP. Cells were grown in complete medium, and NFAT1 expression was determined by immunoblotting with anti-HA. Clones with similar levels of HA-NFAT1 or HA-NFAT1^S79A^ were used for further analysis.

### GFP expression

Forty-eight hours after retroviral transduction of genes in mouse primary T cells or Jurkat cell lines, the cells were washed in FACS buffer (1% BSA plus 0.1% sodium azide) and analyzed by flow cytometry (FACSCalibur using CellQuest Pro 5.2.1 software [BD Biosciences]). Data were analyzed with FlowJo 9.2 software (TreeStar).

### ELISA

IL-2 and TNF-α in culture supernatants were quantitated with Ready-SET-Go ELISA kits (Invitrogen) according to the manufacturer’s instructions. To determine the purity of the pS79-NFAT1 antibody, plates were coated in PBS with 50 μl of a 1 μM concentration of the phosphorylated peptide or nonphosphorylated peptide or blank overnight at room temperature. Plates were washed with PBS-0.05% Tween and blocked with 2% BSA in PBS-0.05% Tween, and antibody was added at different dilutions. Plates were incubated for 1 hour at RT, washed with PBS-0.05% Tween, and incubated with mouse anti-rabbit IgG-HRP. Plates were developed with TMB substrate, the reactions were stopped with 1M H_3_PO_4_, and OD was measured at 405 nm.

### Protein purification

Recombinant p38α, constitutively active MKK6 (S207E/T211E), ATF2, and truncated NFAT1 (tNFAT1, amino acids 1–350) were purified as described [50]. In brief, pET15b vectors containing p38α or MKK6 and pGEX-4T1 vectors containing ATF2 or tNFAT1 were expressed in BL21(DE3) cells. After cultures reached an *A*600 of 0.6–1.0, protein expression was induced with 0.5 mM isopropyl β-D-thiogalactopyranoside (IPTG), and the cultures were incubated overnight at 14 °C. For pET15b vectors containing His-tagged fusion proteins p38α or MKK6, cells were resuspended in binding buffer containing 20 mM Tris pH 7.5, 0.5 M NaCl, 20 mM imidazole, and 1 mM phenylmethylsulfonyl fluoride, sonicated, and centrifuged at 20,000 × *g* for 20 minutes at 4 °C. Proteins were purified with cobalt-charged chelating-Sepharose Fast Flow beads (Amersham Biosciences) and eluted with 0.35 M imidazole in binding buffer. For pGEX-4T1 vectors containing GST-tagged fusion proteins ATF2 or tNFAT1, cells were resuspended in cold PBS and 1 mM phenylmethylsulfonyl fluoride, sonicated, and centrifuged at 20,000 × *g* for 20 minutes at 4 °C. Proteins were purified with Glutathione Sepharose 4 fast Flow beads (GE Healthcare) and eluted with 10 mM reduced glutathione in cold PBS. Proteins were concentrated and washed into water using Microcon YM-30 spin columns (Millipore).

### Real-time PCR

Total RNA from retrovirally transduced mouse T cells was extracted with RNeasy mini kit (Qiagen). After reverse transcription using the Omniscript RT Kit (Qiagen), Power Sybr Green premix (Applied Biosystems) was used for quantitative PCR. All data were normalized to *Hprt* (hypoxanthine-guanine phosphoribosyl transferase) and were presented as relative expression to the background value. The primers used in this study for real-time PCR are as follows: *Nfat2*-Fwd: 5’-GGGTCAGTGTGACCGAAGAT-3’, Rev: 5’-GGAAGTCAGAAGTGGGTGGA-3’; Hprt-Fwd: 5’-AGCCTAAGATGAGCGCAAGT-3’, Rev: 5’-TTACTAGGCAGATGGCCACA-3’.

### In vitro kinase assay

Purified recombinant mouse His-p38α was activated or not by incubation with either 300 ng of active ZAP-70 (R&D systems) or 1 μg of active MKK6 at 30 °C in 20 μl of kinase buffer (20 mM Tris, pH 7.5, 20 mM MgCl_2_, 1 mM dithiothreitol [DTT], 10 mM β-glycerophosphate, and 1 mM Na_3_VO_4_ and 50 μM ATP). After 1 hour, 1 μg ATF2 or tNFAT1 and 10 μCi [^32^P]ATP were added, and incubation proceeded for 45 minutes at 30 °C. Phosphorylated products were resolved by SDS-PAGE, transferred to nitrocellulose membranes, and visualized with a Storm PhosphorImager (GE Healthcare).

### In situ PLA

Duolink in situ PLA enables detection, visualization, and quantification of protein interactions (< 0 nm) as an individual dot by microscopy. Interaction between HA-NFAT1 and calcineurin A was detected in Jurkat cells by PLA using Duolink in Situ detection reagents (Sigma) according to the manufacturer’s protocol with minor modifications. In brief, cells were stimulated with anti-CD3/CD28 or PMA/ionomycin on ibidi 1 μ-slides, fixed with 4% paraformaldehyde, stained with Alexa 488-conjugated wheat germ agglutinin (WGA), permeabilized with methanol, and stained with mouse anti-HA and rabbit anti-calcineurin A primary antibodies. Cells were treated with anti-mouse MINUS and anti-rabbit PLUS probes, ligated, and amplified, and detection was performed by confocal microscopy (Zeiss LSM 880 NLO Airyscan). Slides were analyzed at 80× magnification.

### Confocal microscopy

After stimulation, primary T cells or Jurkat cells were fixed with 4% formaldehyde (Affymetrix) at room temperature for 15 minutes, air-dried on superfrost microscopic glass slides, and blocked with 5% normal goat serum before being incubated overnight with primary antibodies: mouse monoclonal Alexa 647-conjugated anti-HA (6E2, Cell Signaling Technology), anti-NFAT1 (MA1-025, Thermo Scientific), or rabbit polyclonal pS79-NFAT1. Species-matched secondary antibodies conjugated with fluorochrome dye Alexa 594 or Alexa 488 were added for 1 hour. The slides were washed, air-dried, and mounted directly by Prolong Diamond Antifade Mountant with DAPI (P36962, Molecular Probes). Cells stained with only secondary antibodies were used as controls. Images were taken with a Zeiss confocal microscope (Nikon Instruments, Melville, New York, United States), and 10–15 fields/condition were analyzed with ImageJ. PLA data were analyzed by Image-Pro Premier program (version 9.3.1).

### Statistical analysis

*P*-values were calculated by Student *t* test using GraphPad Prism Software.

## Supporting information

S1 Data Data used to generate the manuscript Fig 1A, 1D, 1E, 1F and 1G. (XLSX)

S2 Data Data used to generate the manuscript Fig 2C. (XLSX)

S3 Data Data used to generate the manuscript Fig 3D. (XLSX)

S4 Data Data used to generate the manuscript Fig 4A and 4D. (XLSX)

S5 Data Data used to generate the manuscript Fig 5D. (XLSX)

S6 Data Data used to generate the supplemental S4 Fig. (XLS)

S1 Fig T-cell antigen receptor (TCR)-stimulated p38 pY323 uniquely up-regulates c-Fos expression. Purified T cells from wild-type (WT) or double knock-in (DKI) mice were stimulated with anti-CD3/CD28 for the indicated times, and the lysates were immunoblotted for c-Fos and c-Jun expression.
(TIF)

S2 Fig Infection efficiency of purified primary T cells from wild-type (WT) and double knock-in (DKI) mice. Purified T cells from WT or DKI mice were stimulated with anti-CD3/CD28, transduced with retrovirus encoding empty vector (EV) or c-Fos, and then stimulated with anti-CD3/CD28 for 48 hours. Infection efficiency was determined by flow cytometric measurement of green fluorescent protein (GFP) expression (A). Purified T cells from WT mice were stimulated and infected as in panel A, and GFP expression was assessed by flow cytometry (B).
(TIF)

S3 Fig Unique NFAT1^S79^ phosphorylation by zeta-associated protein (ZAP-70)-activated p38. Recombinant mouse p38α was incubated with active human ZAP-70 or mitogen-activated protein kinase kinase 6 (MKK6) and recombinant tNFAT1 as substrate, followed by mass spectrometry. The results are representative of 2 independent experiments.
(TIF)

S4 Fig Specificity of anti-pNFAT1^S79A^. ELISA plates were coated with 50 μl of PBS alone or containing the immunizing NFAT1 peptide either unphosphorylated or phosphorylated at S79 at a concentration of 1 μM overnight at room temperature. Plates were blocked with 2% BSA-PBS-0.05% Tween and then incubated with the indicated concentrations of the column-purified anti-NFAT1-S79A antibody. Plates were developed with rabbit immunoglobulin G (IgG)-horseradish peroxidase (HRP) antibody followed by incubation with TMB substrate and quantitation with an ELISA reader (S6 Data).
(TIF)

S5 Fig CD3 and T-cell antigen receptor (TCR)-β expression in wild-type (WT) and N1KO Jurkat cells. Flow cytometric measurement of surface CD3 and TCR-β expression on Jurkat cells and subclones in which NFAT1 was disrupted.
(TIF)

S6 Fig Retroviral transduction of Jurkat cells with HA-NFAT and HA-NFAT1^S79A^. Jurkat cells were infected with retrovirus encoding HA-NFAT1 or HA-NFAT1-S79A, and after 72 hours, the infection efficiency was assessed by flow cytometry for green fluorescent protein (GFP) expression (A). Jurkat cells were infected as in panel A and stimulated with anti-CD3/CD28 for 3 hours, and NFAT1 (red) localization was assessed by confocal microscopy (B). Jurkat cells were infected as in panel A and stimulated with anti-CD3/CD28 for 3 hours, and NFAT1 localization was assessed by immunoblotting cytosolic and nuclear fractions (C). Purified T cells from wild-type (WT) mice were infected with retrovirus and stimulated with anti-CD3/CD28 for 1 hour, and the infection efficiency was assessed by flow cytometry for GFP expression (D). Jurkat cell lines expressing WT-NFAT1 or NFAT1^S79A^ were stimulated with anti-CD3/CD28 and lysed, and calcineurin A and HA-NFAT1 levels were quantitated by immunoblotting (E).
(TIF)

S1 Table Recombinant mouse p38α was incubated with active human zeta-associated protein (ZAP-70) or mitogen-activated protein kinase kinase 6 (MKK6) in in vitro kinase buffer. After 1 hour, recombinant tNFAT1 was added and incubated for an additional hour before analysis by mass spectrometry on an Oribitrap Fusion. Data were analyzed by Proteome Discoverer. The table shows the peptide sequences identified to be phosphorylated, the site of phosphorylation, the number of peptide spectral matches per peptide, and related statistics of peptide matching confidence.
(XLSX)

## Abbreviations

ATF2: activating transcription factor 2
ChIP: chromatin immunoprecipitation
DKI: double knock-in
EAE: experimental autoimmune encephalomyelitis
eGFP: enhanced green fluorescent protein
ERK: extracellular signal-regulated kinase
EV: empty vector
FasL: Fas ligand
GM-CSF: granulocyte-macrophage colony-stimulating factor
HPF: high power field
IFN-γ: interferon gamma
IL: interleukin
IRES: internal ribosome entry site
JNK: c-Jun N-terminal kinase
MAPK: mitogen-activated protein kinase
MEF-2: myocyte enhancer factor-2
MIF-1α: migration inhibitory factor 1 alpha
MKK6: mitogen-activated protein kinase kinase 6
MOG: myelin oligodendrocyte glycoprotein
NFAT: nuclear factor of activated T cells
NIH: National Institutes of Health
NLS: nuclear localization signal
PLA: proximity ligation assay
PMA: phorbol myristate acetate
qRT-PCR: real-time quantitative reverse transcription PCR
RANKL: receptor activator of nuclear factor κB ligand
S/T: serine/threonine
siRNA: small interfering RNA
TCR: T-cell antigen receptor
TNF-α: tumor necrosis factor alpha
TonEBP: tonicity-responsive enhancer binding protein
WGA: wheat germ agglutinin
WT: wild type
ZAP-70: zeta-associated protein
