# Supplementary figures and images for: Unique properties of TCR-activated p38 are necessary for NFAT-dependent T-cell activation
Source: PLoS Biol. 2018 Jan 22;16(1):e2004111. doi: 10.1371/journal.pbio.2004111 (PMC5794172; doi:10.1371/journal.pbio.2004111)

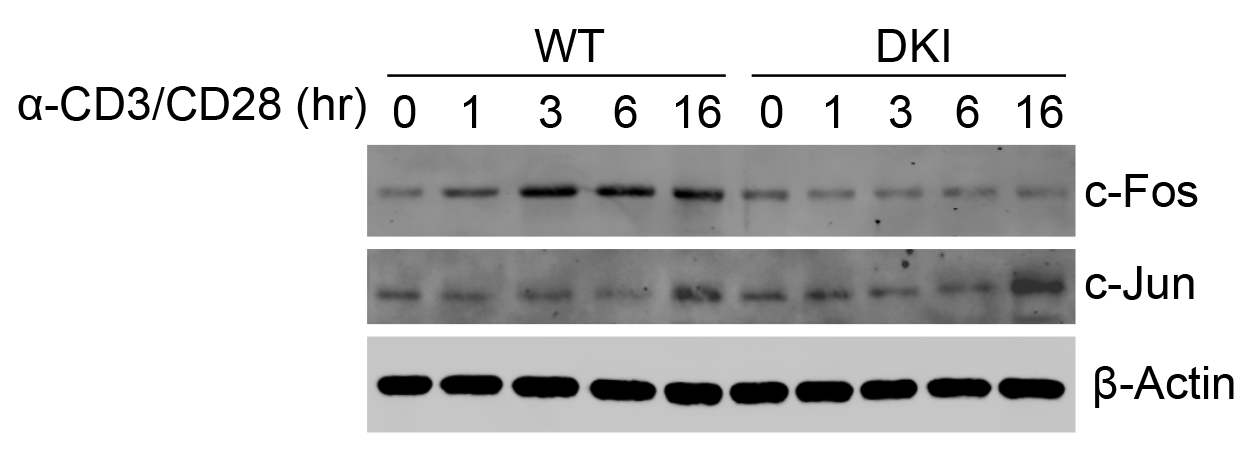

Supplement: S1 Fig — Purified T cells from wild-type (WT) or double knock-in (DKI) mice were stimulated with anti-CD3/CD28 for the indicated times, and the lysates were immunoblotted for c-Fos and c-Jun expression. (TIF) [file pbio.2004111.s007.tif]

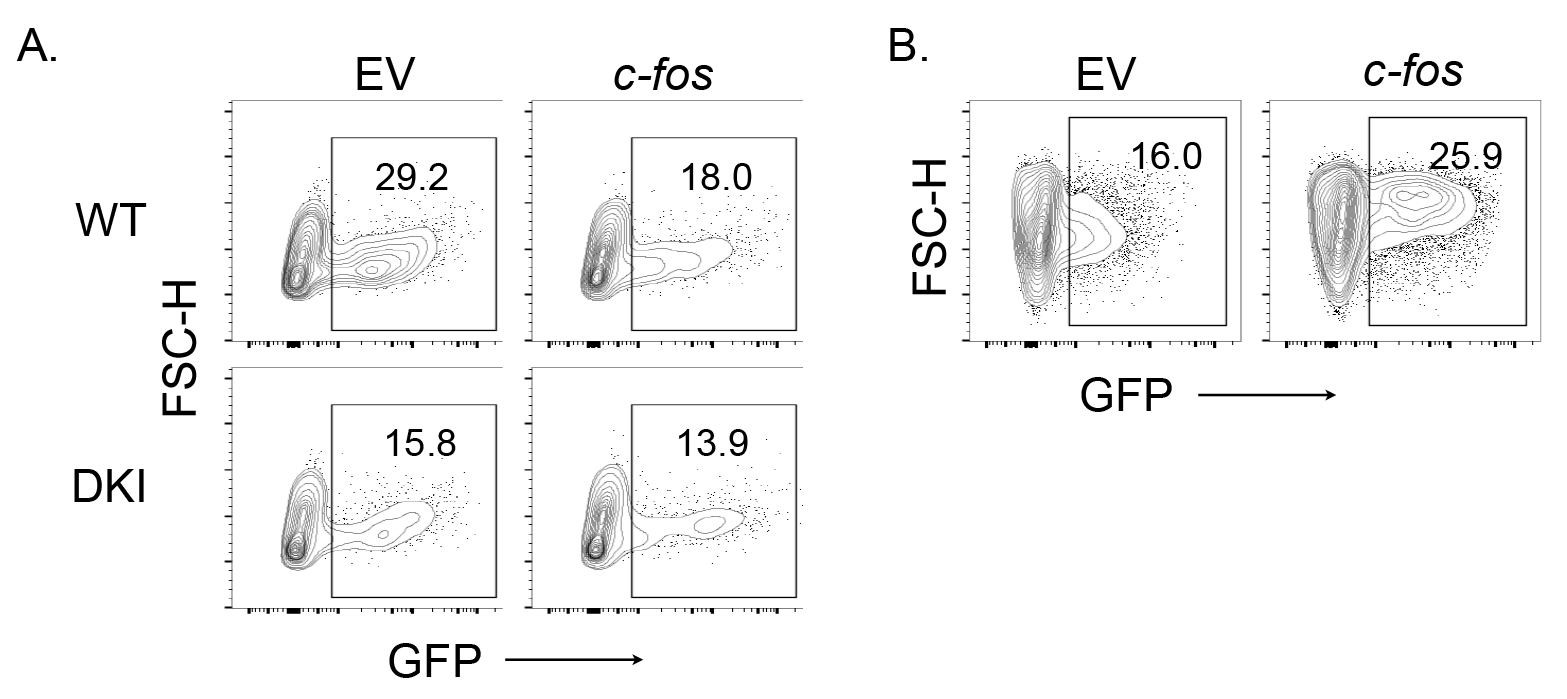

Supplement: S2 Fig — Purified T cells from WT or DKI mice were stimulated with anti-CD3/CD28, transduced with retrovirus encoding empty vector (EV) or c-Fos, and then stimulated with anti-CD3/CD28 for 48 hours. Infection efficiency was determined by flow cytometric measurement of green fluorescent protein (GFP) expression (A). Purified T cells from WT mice were stimulated and infected as in panel A, and GFP expression was assessed by flow cytometry (B). (TIF) [file pbio.2004111.s008.tif]

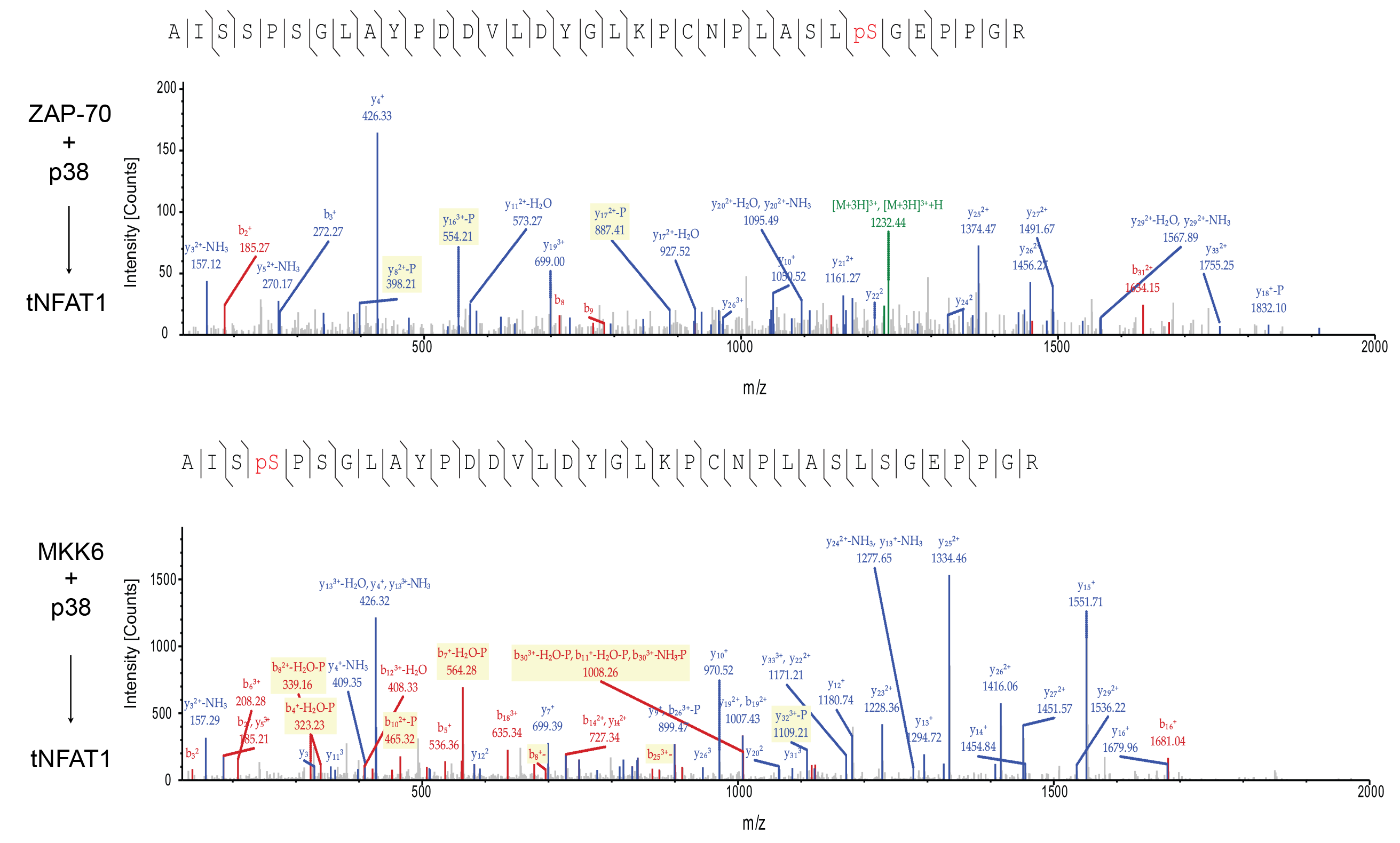

Supplement: S3 Fig — Recombinant mouse p38α was incubated with active human ZAP-70 or mitogen-activated protein kinase kinase 6 (MKK6) and recombinant tNFAT1 as substrate, followed by mass spectrometry. The results are representative of 2 independent experiments. (TIF) [file pbio.2004111.s009.tif]

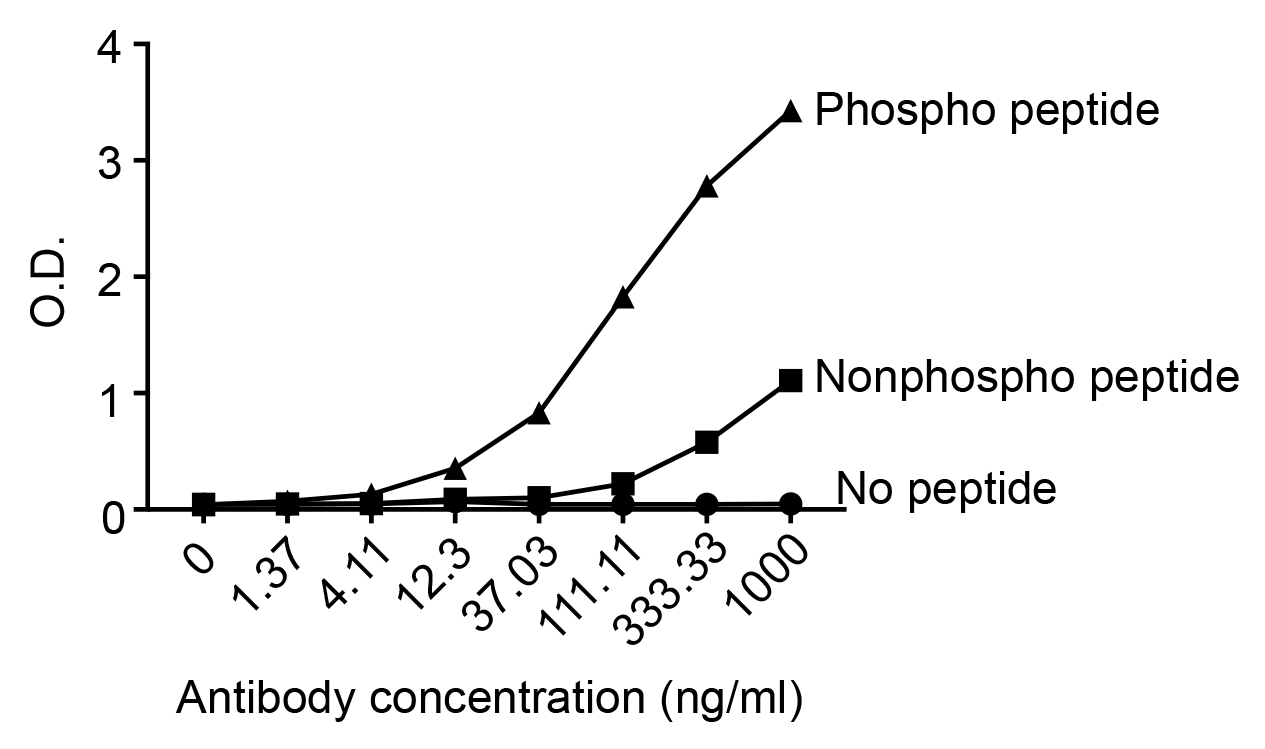

Supplement: S4 Fig — ELISA plates were coated with 50 μl of PBS alone or containing the immunizing NFAT1 peptide either unphosphorylated or phosphorylated at S79 at a concentration of 1 μM overnight at room temperature. Plates were blocked with 2% BSA-PBS-0.05% Tween and then incubated with the indicated concentrations of the column-purified anti-NFAT1-S79A antibody. Plates were developed with rabbit immunoglobulin G (IgG)-horseradish peroxidase (HRP) antibody followed by incubation with TMB substrate and quantitation with an ELISA reader (S6 Data). (TIF) [file pbio.2004111.s010.tif]

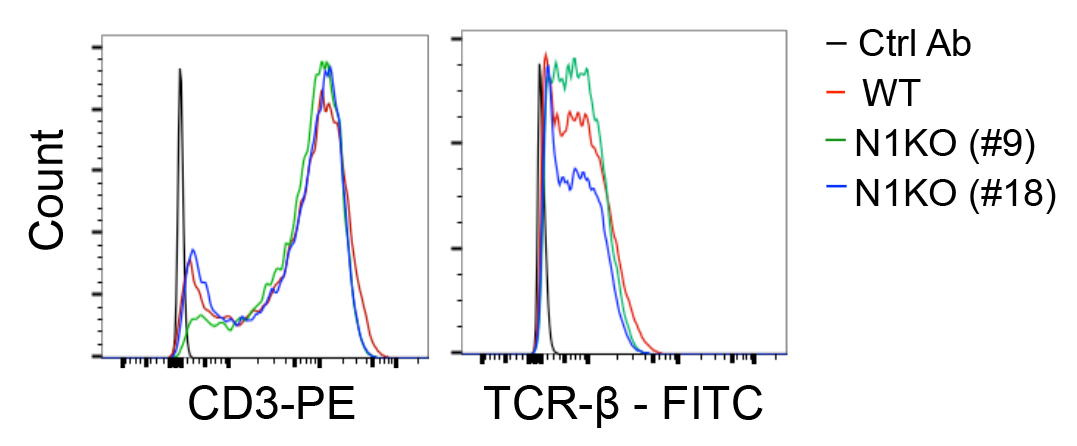

Supplement: S5 Fig — Flow cytometric measurement of surface CD3 and TCR-β expression on Jurkat cells and subclones in which NFAT1 was disrupted. (TIF) [file pbio.2004111.s011.tif]

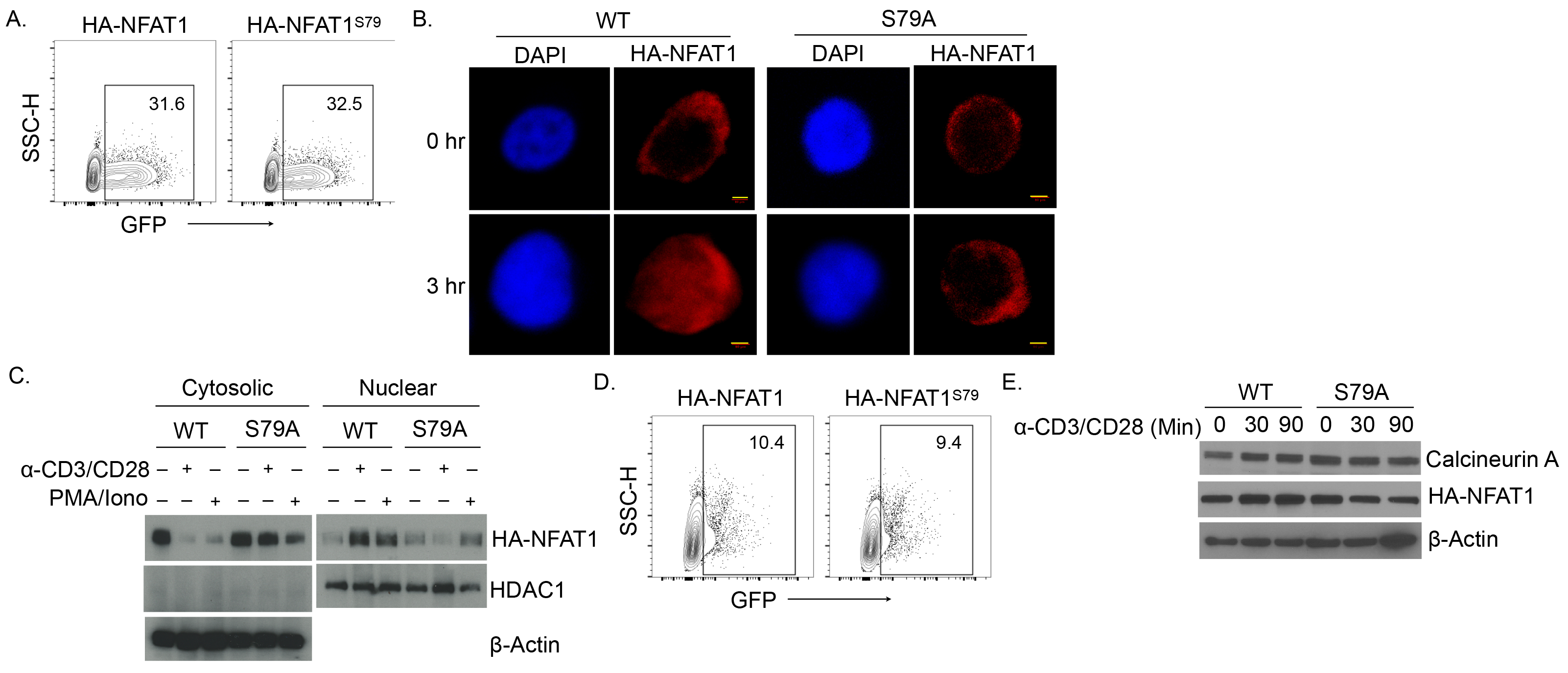

Supplement: S6 Fig — Jurkat cells were infected with retrovirus encoding HA-NFAT1 or HA-NFAT1-S79A, and after 72 hours, the infection efficiency was assessed by flow cytometry for green fluorescent protein (GFP) expression (A). Jurkat cells were infected as in panel A and stimulated with anti-CD3/CD28 for 3 hours, and NFAT1 (red) localization was assessed by confocal microscopy (B). Jurkat cells were infected as in panel A and stimulated with anti-CD3/CD28 for 3 hours, and NFAT1 localization was assessed by immunoblotting cytosolic and nuclear fractions (C). Purified T cells from wild-type (WT) mice were infected with retrovirus and stimulated with anti-CD3/CD28 for 1 hour, and the infection efficiency was assessed by flow cytometry for GFP expression (D). Jurkat cell lines expressing WT-NFAT1 or NFAT1S79A were stimulated with anti-CD3/CD28 and lysed, and calcineurin A and HA-NFAT1 levels were quantitated by immunoblotting (E). (TIF) [file pbio.2004111.s012.tif]
